# Identification of differential plasma miRNA profiles in Chinese workers with occupational lead exposure

**DOI:** 10.1042/BSR20171111

**Published:** 2017-10-31

**Authors:** Ming Xu, Zhengmin Yu, Feifei Hu, Hongbing Zhang, Lixin Zhong, Lei Han, Yan An, Baoli Zhu, Hengdong Zhang

**Affiliations:** 1 Department of Occupational Disease Prevention, Jiangsu Provincial Center for Disease Control and Prevention, Nanjing 210009, China; 2 Changzhou Center for Disease Control and Prevention, Changzhou 213022, China; 3 Department of Toxicology, School of Public Health, Medical College of Soochow University, Suzhou 215123, China

**Keywords:** blood lead level, chronic lead exposure, diagnosis, microRNA

## Abstract

Elevated lead absorptions are hazardous factors in lead-related workers. Previous studies have found its toxic impacts on nervous, circulatory, and metabolic systems. We hypothesized that alteration of miRNAs profile in plasma was closely associated with lead exposure. We analyzed to identify lead-related miRNAs in workers occupationally exposed to lead. Microarray assay was performed to detect plasma miRNA between workers with high and minimal lead exposure in the discovery stage. The following prediction of miRNAs’ candidate target genes was carried out by using miRecords, STRING, and KEGG databases. We finally identified four miRNAs significantly associated with high level of blood lead. *miR-520c-3p* (*P=0.014), *miR-211* (*P=0.019), and *miR-148a* (*P=0.031) were downexpressed in workers with high lead exposure and with high blood lead level (BLL), while *miR-572*(*P=0.027) displayed an opposite profile. Functional analysis of miRNAs displayed that these miRNAs could trigger different cellular genes and pathways. People under chronic lead exposure had a diverse ‘fingerprint’ plasma miRNA profile. Our study suggested that *miR-520c-3p, miR-211, miR-148a*, and *miR-572* were the potential biomarkers for lead susceptibility in Chinese.

## Introduction

Lead (Pb) is a common material existing in the Earth, and widely utilized in industry. The most important artificial sources for lead emission are considered to be mining and metal smelting [1,2]. As a classical environmental and occupational toxicant, it could cause a series of severity diseases, involved in hemopoietic, nervous, digestive, urinary, and even reproductive systems. In recent research, lead was believed to cause direct DNA damage and to be associated with renal cell cancer (RCC) [3,4]. In 2006, the inorganic lead compounds were considered as potentially carcinogenic to humans by IARC organization [5].

With economy blooming, both governments and workers have become more aware of the lead-induced occupational health problems. The U.S. National Institute of Occupational Safety and Health (NIOSH) report estimated that over 3 million workers in U.S.A. were potentially exposed to lead during their working time [6]. In 2014, the Chinese Center for Disease Control and Prevention (CDC) reported 224 occupational disease cases with chronic lead poisoning, with over 600 μg/dl blood lead levels (BLLs) based on current diagnostic criteria of occupational diseases in China. The main industrial exposure sources for lead poisoning include battery recycling, lead-induced gasoline industry, bearing arm working, pipes manufacturing, boat building, and printing [7]. Children, living with lead-related patients, might also suffer from lead exposure by pinning on lead from patients’ clothes or skin [8]. Besides, agricultural soil close to these industrial factories is also vulnerable to pollution by flooding or irrigation, especially in the downstream areas. Lead contamination has been commonly detected in plants, livestock, poultry, and humans consuming these products.

Lead, along with many other toxic metals, is closely associated with epigenetic modifications in humans, including DNA methylation, histone deacetylation, and miRNA dysregulation [9–11]. These epigenetic changes might also influence gene expression by various mechanisms. As an important component of epigenetics, miRNAs are described as a group of small non-coding RNAs with approximately 22-bp length. These non-coding RNAs usually perform their functions by binding to the 3′-UTRs of their target genes’ mRNA, and interfere with the translation of these mRNAs.

Till now, the recent researches have comprehensively investigated the profiles of miRNAs after exposure to lead in different organs [12–14], which partly revealed the mechanism of this lead-induced miRNAs and suggested their impacts. However, no research on miRNA has been conducted on the susceptibility of lead exposure. In the present study, we sought to investigate the different miRNAs existed in highly internal lead-exposed persons opposite to those minimally internal lead exposed, and organize these results to serve as a potential diagnosis biomarker for lead-exposed workers.

## Materials and methods

### Study areas and samples

The present study was approved by the Ethics Committee of Jiangsu Provincial CDC, Nanjing, China (approval number: 2012025) and the corresponding methods were carried out in accordance with the approved guidelines. All participating workers had been informed about the content of this research and signed the written informed consents before donating their blood samples.

A total of 1213 participants were enrolled. They were from five battery factories in different administrative regions of Jiangsu Province, China, since January 2004. All the five battery factories we chose were large-scale factories located in the northern part of Jiangsu Province, which were far from the cities and towns (at least 10 kilometers away), and no other factory was within 5 kilometers. The employees were usually enrolled from the relatively nearby fixed towns, with similar lifestyles. These participants experienced similar external lead exposure dose (C_TWA_ =0.025 ± 0.009 mg/m^3^) during work. In their health examination during work orientation, we excluded participants with a history of hematological disorder, liver or kidney dysfunction, or with exposure to lead-containing medical therapy in their daily lives. Following the guide of trained staff, each participant completed a standard questionnaire, including demographic information, detailed occupational history, medical history, individual habits, and self-consciousness symptoms. In education situation, illiterate meant that participant did not complete primary school, literate and up to lower secondary level meant that participant completed primary but not junior high school, low up to middle secondary level indicated that participant finished junior high but dropped out of senior high school, and the higher secondary level and above indicated that participant completed at least senior high school. In eat or drink in workshop, participants who never had lunch or dinner at workplace belonged to the group ‘No’, those who ate no more than once a week at workplace were defined as occasional, and all the others were categorized as ‘Yes’. Blood samples of participants were taken in annual physical examination and stored at −80°C for further analyses. As an occupational disease study, we did not enroll healthy people without lead exposure as controls, because lead had been considered as the dominant predisposing factor and it was unnecessary and not cost effective to enroll control group. Instead, top 10% participants with the highest BLL and bottom 10% participants with the lowest BLL were defined as high and minimal lead-exposure groups, respectively, in the present study.

### RNA extraction and purification

Total RNA from blood was extracted by miRNeasy Serum/Plasma Kit (Qiagen, Germany) according to manufacturer’s protocol to reach appropriate purity for further microarray and quantitative reverse-transcriptase PCR (qRT-PCR). Nanodrop One^C^ (Thermo, Waltham, U.S.A.) was adopted to measure the quality and quantity of these RNA samples. All RNA samples were stored at −80°C for further usage.

For preparation of microarray, a high and a minimal exposure plasma pool were prepared, each group contained ten samples to detect the most significant discrepant miRNAs, as described by our previous studies [7,15]. Cel-*miR-238* was added into each plasma sample as the internal control in real-time PCR for validation.

### MiRNA array profiling

Approximately 4–8 μg purified total RNA samples were used for microarray, which were labeled with 3′-extended poly(A) tail structure as pretreatment. By binding to these poly(A) tails, an oligonucleotide tag could closely ligate with miRNAs, which were essential for subsequent fluorescent dying. The following RNA hybridization was carried out by μParaflo® microfluidic microarray (Atactic Technologies) [16]. Each detection probe contained a chemically modified nucleotide coding segment complementary to the target miRNAs (reported in miR base, http://www.miRbase.org/, and/or customer-defined sequences) and a segment of PEG to extend the coding segment away from the substrate. The tag-conjugating Cy3 dyes were circulated from microfluidic microarray for dye staining. Fluorescence images were collected using a laser scanner, converted into digital images, and then processed with Array-Pro Image Analysis Software (Media Cybernetics Inc, Rockville, U.S.A.). The final data were obtained by subtracting the background and normalizing the signals using a locally weighted regression filter as described [16].

Screening criteria of microarray for further validation were as follows: (i) miRNAs were expressed differently (up-regulated or down-regulated) between high and minimal lead-exposure groups; (ii) demonstrated at least a 2-fold increase or 0.5-fold decrease in high lead-exposure group compared with minimal exposure group; (iii) at least 500 copies in each of the two groups.

### MiRNA expression

qRT-PCR was performed to measure the consequences of candidate miRNAs, which were selected in the above microarray. The miRNA-specific stem-loop primers and Taqman miRNA Reverse Transcription Kit (Applied Biosystems, U.S.A.) were used in reverse-transcription step according to the manufacturer, and final real-time PCR was performed by ABI 7900HT Fast Real-Time PCR System (Applied Biosystems) with their specific primers (Applied Biosystems) against a cel-*miR-238* internal control. All PCR reactions were triplicated to ensure the reliability of candidate miRNAs’ expression in each sample. In order to eliminate the miRNA degradation and the operating error, the detection of serum miRNAs in 113 high and 113 minimal lead-exposure groups was completely performed in 5 days by three experienced operators.

### Prediction and functional analysis of target genes

The target genes of miRNAs were predicted in miRecords database (http://c1.accurascience.com/miRecords/prediction_query.php), which is an integration platform of miRNA target prediction composed by DIANA-microT, MicroInspector, miRanda, MirTarget, miTarget, NBmiR Tar, Pic Tar, PITA, RNA22, RNAhybrid, and TargetScan/TargetScans programs. Functional analysis of these predicted target genes was performed in STRING database (https://string-db.org/) and KEGG database (http://www.genome.jp/kegg/pathway.html).

### Statistical analysis

Statistical analyses were carried out by SAS Software (version 10.0, SAS Institute Inc, Cary, U.S.A.). Distinctions between high and minimal internal lead-exposure groups were detected by χ^2^ test without special explanations. Student’s *t* tests were performed for age, BLL, and different expressions of various miRNA involved in the present study. All *P*-values were two-sided with *P*<0.05 as statistically significant.

## Results

### Characteristics of study participants

The complete information on the final 1130 participants is shown in Table 1, including gender, age, marital status, educational background, smoking and alcohol consumption, eating and drinking behavior at work, and BLL. The majority of these workers were in the age of 30–50 years (79.12%), married (98.49%), and received the 9-year compulsory education in China (59.82%); 301 (26.64%) and 313 (27.70%) participants were smokers and drinkers, respectively; 448 (39.65%) workers usually had lunch or dinner in their workplace, comparing those with occasional behavior (26.28%) and those without this habit (33.53%). The latest BLLs of these workers were 386.73 ± 177.93 μg/l, ranging from 17 to 1060 μg/l.

**Table 1:** Demographic characters and BLLs of all the participants

Participant characteristics	*n*=1130
	*n* (%)
**Gender**	
Male	599 (53.0)
Female	531 (47.0)
**Age (years)**	
(20, 30)	83 (7.4)
(30, 40)	275 (24.3)
(40, 50)	619 (54.8)
(50, 60)	136 (12.0)
(60, 70)	17 (1.5)
**Marriage**	
Single	3 (0.2)
Married	1113 (98.5)
Divorced	14 (1.3)
**Education**	
Illiterate	67 (5.9)
Literate and up to lower secondary level	158 (14.0)
Low up to middle secondary level	676 (59.8)
Higher secondary level and above	229 (20.3)
**Smoking**	
No	829 (73.4)
Yes	301 (26.6)
**Drinking**	
No	817 (72.3)
Yes	313 (27.7)
**Eat or drink in workshop**	
No	379 (33.5)
Occasionally	303 (26.8)
Yes	448 (39.7)
**BLL (μg/l)**	
Mean ± S.D.	386.73 ± 177.93 (17–1060)

The characteristics of the high and minimal lead-exposure groups are shown in Table 2. Besides BLL (*P*<0.001), there was marginally significant difference in age (*P*=0.047) as well. There were no significant differences in gender, education, smoking, drinking, and eating habits between these two groups (*P*≥0.05).

**Table 2:** The characters of 10% lead-sensitive group and 10% lead-resistant group

Characteristics	Group		*P*
	Lead resistant (*n*=113) *n* (%)	Lead sensitive (*n*=113) *n* (%)	
**Gender**			0.506
Male	52 (46.0)	57 (50.4)	
Female	61 (54.0)	56 (49.6)	
**Age (years)**	35.86 ± 10.26	38.39 ± 8.85	0.047*
**BMI (kg/m^2^)**	23.7 ± 3.6	24.3 ± 4.8	0.289
**Smoking**			0.246
No	83 (73.4)	75 (66.4)	
Yes	30 (26.6)	38 (33.6)	
**Education**			0.412
Literate and up to lower secondary level	21 (18.6)	26 (23.0)	
Low up to middle secondary level	92 (81.4)	87 (77.0)	
**Drinking**			0.080
No	93 (82.3)	82 (72.6)	
Yes	20 (17.7)	31 (27.4)	
**Eat or drink in workplace**			0.847
No	31 (27.4)	30 (26.6)	
Occasionally	35 (31.0)	39 (34.5)	
Yes	47 (41.6)	44 (38.9)	
BLL (μg/l)*			<0.001*
Mean ± S.D.	89.34 ± 15.39	513.52 ± 63.86	

* *P*-value of two-sided Student’s *t* test for age and BLL. Abbreviation: BMI, body mass index.

**Table 3:** The expression levels of selected human miRNAs in mircoarray

miRNA	Discovery stage		Trend	FC
	Lead resistant	Lead sensitive		
hsa-*miR-520c-3p*	43217	12490	Up	5.41
hsa-*miR-211*	16018	4630	Up	3.46
hsa-*miR-148a*	10894	4323	Up	2.52
hsa-*miR-141*	2068	953	Up	2.17
hsa-*miR-572*	513	1140	Down	0.39
hsa-*miR-130b*	4146	10630	Down	0.45

Abbreviation: FC, fold change.

### Differentially expressed plasma miRNAs between chronic high and minimal internal lead-exposed workers

**Figure 1:**
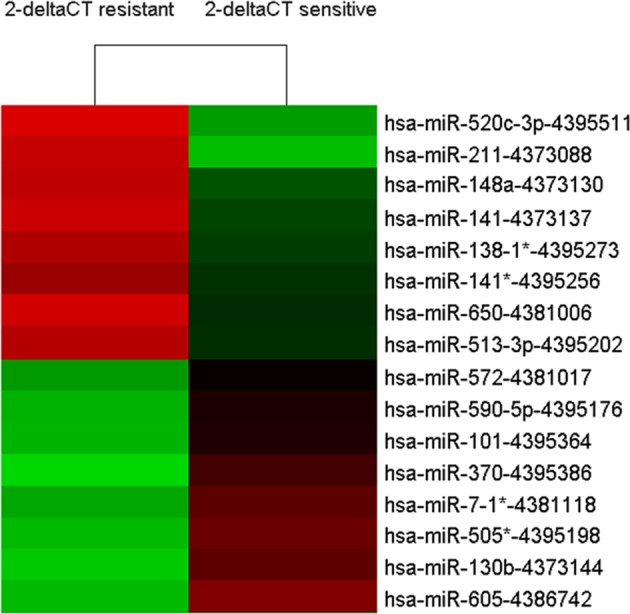
Differentially expressed miRNAs between highly internal lead-exposed and minimally internal lead-exposed workers in microarray. Fold change ≥2.0 The red color indicates up-regulated miRNAs and the green color indicates down-regulated miRNAs. The symbol (*) represents the miRNA minor.

The results of microarray in highly and minimally internal lead-exposed groups for miRNA prolife detection are shown in Figure 1 and Table 3. Finally, four down-regulated miRNAs (hsa-*miR-520c-3p*, hsa-*miR-148a*, hsa-*miR-141*, and hsa-*miR-211*) and two up-regulated miRNAs (hsa-*miR-572* and hsa-*miR-130b*) were selected based on the screening criteria.

### Lead-induced miRNA expression was associated with chronic lead exposure

**Figure 2:**
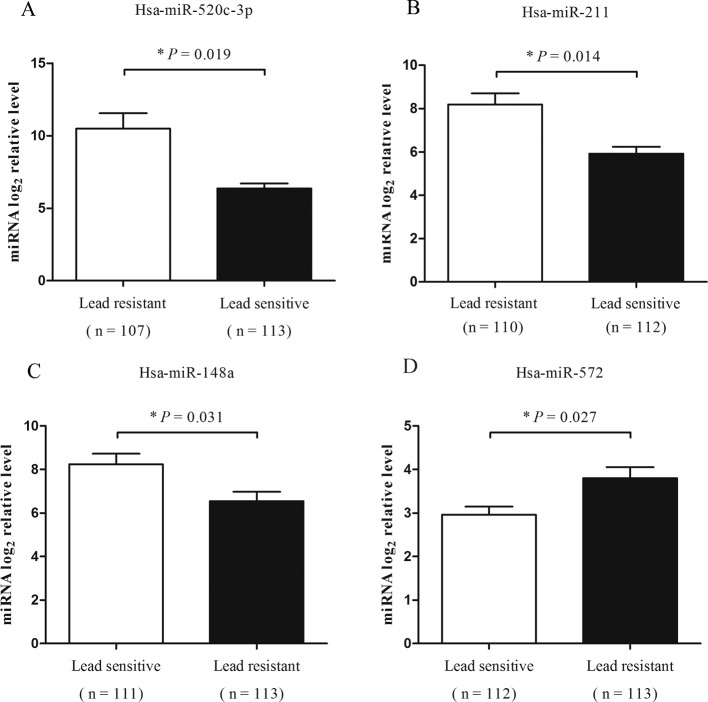
Significant plasma expressions of testing miRNAs in two groups (**A**) *miR-520c-3p* profile; (**B**) *miR-211* profile; (**C**) *miR-148a* profile; (**D**) *miR-572* profile. *P: *P*-value adjusted for sex, age, BMI, smoking, and education, drinking, and eating habits in workplace.

To further validate whether the above miRNAs were actually associated with chronic lead exposure and could be potential lead exposure susceptibility biomarkers, we then analyzed these miRNAs in the samples of high and minimal internal lead-exposure groups, respectively. In Figure 2, compared with the minimal internal lead-exposure group, *miR-520c-3p, miR-211*, and *miR-148a* were significantly lower (*P=0.019, 0.014, and 0.031, respectively) while *miR-572* were significantly higher in the high internal lead-exposure group (*P=0.027). These results were in accordance with their expression in microarray analysis well. However, *miR-141* and *miR-130b* showed no significant differences between these two groups (*P>0.05).

### Functional analysis of miRNAs

**Figure 3:**
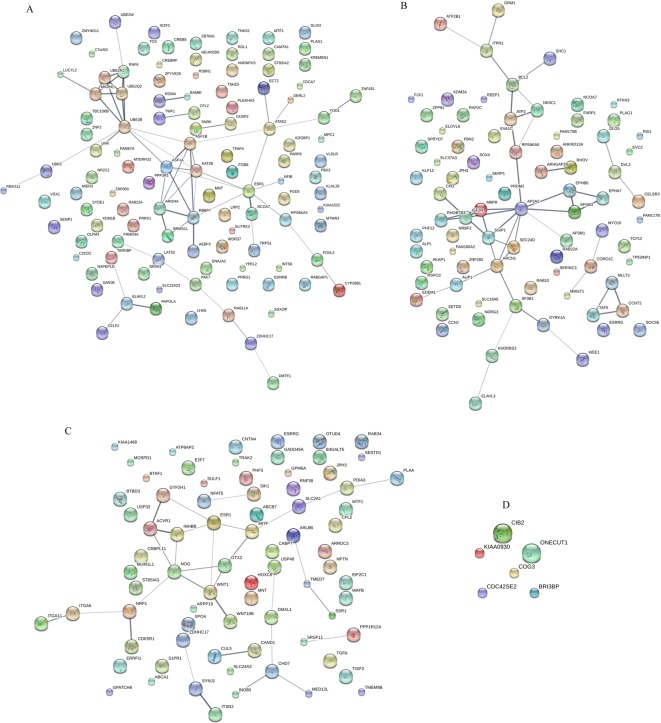
Functional analysis of miRNAs’ target genes (**A**) *miR-520c-3p*’s target genes; (**B**) *miR-211*’s target genes; (**C**) *miR-148a*’s target genes; and (**D**) *miR-572*’s target genes.

We further predicted the target proteins of these miRNAs. *miR-520c-3p, miR-211*, and *miR-148a* had 110, 80, and 76 potential candidate genes, respectively. *miR-572* had only six candidates (Table 4). These target genes constituted an interacting network and pathway, which included cell proliferation, apoptosis, motility, and even survival. In the miRecords data, we chose to enroll the genes predicted by six programs for *miR-148a* and *miR-211*, and genes predicted by five programs for *miR-520c-3p* and *miR-572* (there was no candidate gene for these two miRNAs when they were predicted by six programs simultaneously). And we performed the following functional analysis of these predicted targets of *miR-520c-3p, miR-211, miR-148a*, and *miR-572*, respectively (Figure 3). *miR-520* might be involved in the SUMOlation pathway, which was a novel modification of protein in eukaryotic cellular processes. *miR-211* could possibly trigger cellular apoptosis by regulating Bcl-2 signal pathway, and influence phagocytosis by targetting the M6PR pathway. *miR-148a* could potentially invoke the endoplasmic reticulum stress by targetting phospholipase A2 activating protein (PLAA) and its corresponding downstream genes. *miR-148a* might also regulate the microphthalmia-associated transcription factor (MITF) pathway in osteoclasts, and eventually impacted osteoclasts differentiation. *miR-572*, however, did not match any important signal pathway in our analysis. Although the majority of the predicted target genes of these miRNAs need further validation, these initial target genes could reveal how these miRNAs mediated different pathways related to diseases by lead exposure.

**Table 4:** Prediction of target genes for *miR-572, miR-211, miR-520-3p*, and *miR-148a*

Target genes of miRNAs							
*miR-572*	*miR-211*		*miR-520c-3p*		*miR-148a*		
*C22orf9*	*NCOA7*	*AP3M1*	*SLITRK3*	*FGD5*	*KREMEN1*	*E2F7*	*CDK5R1*
*CDC42SE2*	*RAB10*	*ARCN1*	*ASF1B*	*OLFM3*	*DUSP2*	*SLC24A3*	*NRP1*
*ONECUT1*	*PID1*	*ATP2B1*	*C7orf43*	*SYDE1*	*ELAVL2*	*JPH3*	*CUL5*
*CIB2*	*CCNJ*	*ATF2*	*RSBN1*	*CFL2*	*LRP2*	*TMEM9B*	*WNT10B*
*COG3*	*SETD8*	*FBN2*	*YOD1*	*SLC22A23*	*HNRNPH3*	*MNT*	*TGFA*
*BRI3BP*	*C13orf1*	*ITPR1*	*PLEKHA3*	*NEUROD6*	*BAMBI*	*MOSPD1*	*SSR1*
	*JPH3*	*M6PR*	*CYP26B1*	*FOXL2*	*MKRN1*	*ERRFI1*	*PPP1R12A*
	*RAB22A*	*PLAG1*	*RGMA*	*PARP8*	*SSX2IP*	*CAND1*	*ITGA5*
	*RAP2C*	*SHC1*	*MNT*	*FAM57A*	*WDR37*	*ARL8B*	*INHBB*
	*RHOBTB3*	*ESRRG*	*PAK7*	*FBXO11*	*ASF1A*	*CHD7*	*GADD45A*
	*SEC24D*	*ELAVL3*	*RAB22A*	*TNKS2*	*ATAD2*	*INOC1*	*ESRRG*
	*AP2A2*	*ALPL*	*TSHZ3*	*CDCA7*	*TRPS1*	*ST8SIA3*	*S1PR1*
	*FBXL11*	*BCL2*	*ECT2*	*BRMS1L*	*SENP1*	*ZDHHC17*	*ACVR1*
	*MYO10*	*GRM1*	*FRMD4A*	*PAPOLA*	*LATS2*	*SULF1*	*ITGA11*
	*SF3B1*	*IGF2R*	*UBE2R2*	*LHX6*	*VSX1*	*PHF3*	*GPATCH8*
	*CORO1C*	*CCNT2*	*RGL1*	*EDNRB*	*RABGAP1*	*TRAK2*	*MITF*
	*FJX1*	*CPD*	*CAMTA1*	*IKZF2*	*TARDBP*	*USP33*	*BTAF1*
	*SERP1*	*EFNB3*	*ZDHHC17*	*UNK*	*NR4A3*	*BTBD3*	*PLAA*
	*EDEM1*	*CELSR3*	*ZFYVE26*	*ITGB8*	*RPS6KA3*	*ARRDC3*	*ARPP-19*
	*NDRG3*	*SOX4*	*C2CD2*	*PLAG1*	*RAB11A*	*KIAA1468*	*NFAT5*
	*REEP1*	*TCF12*	*LUC7L2*	*TWF1*	*NFIB*	*OTUD4*	*SLC2A1*
	*C21orf63*	*RPS6KA5*	*DERL2*	*ARID4A*	*DNAJA2*	*CABP7*	*ITSN2*
	*DYRK1A*	*ARHGAP29*	*BRP44L*	*RBBP7*	*MTF1*	*TMED7*	*MTF1*
	*RTKN2*	*FARP1*	*KLHL28*	*TFAP4*	*RNF6*	*SESTD1*	*ATP6AP2*
	*EVC2*	*KHDRBS3*	*SNRK*	*NR2C2*	*ST8SIA2*	*CNTN4*	*DMXL1*
	*PHF13*	*SERINC3*	*TBC1D8B*	*UBE2B*	*PBX3*	*SNF1LK*	*ABCA1*
	*TMEM32*	*TAF5*	*KIAA1522*	*PCAF*	*IGF2BP1*	*MUM1L1*	*MAFB*
	*ALS2CR13*	*CHP*	*MTMR3*	*ZNF436*	*ZMYND11*	*MED12L*	*NOG*
	*AUP1*	*KLF12*	*DMTF1*	*GLIS3*	*PRRX1*	*USP48*	*WNT1*
	*ZFP91*	*DLG5*	*MIER3*	*INTS6*	*TOX*	*RAB34*	*GTF2H1*
	*TP53INP1*	*SLC16A6*	*AOF1*	*ESR1*		*RNF38*	*PDIA3*
	*ELOVL6*	*WEE1*	*AEBP2*	*PPP3R1*		*OSBPL11*	*GPM6A*
	*NRBF2*	*AKAP1*	*C5orf41*	*PRRG1*		*HOXC8*	*B4GALT5*
	*FAM160A2*	*ZNF282*	*UBR3*	*UBE2W*		*XPO4*	*SFRS11*
	*KIAA0157*	*SOCS6*	*UBE2Q2*	*YPEL2*		*CFL2*	*ABCB7*
	*SGIP1*	*DVL3*	*ZNF800*	*CREB5*		*TGIF2*	*ESR1*
	*SLC37A3*	*EPHA7*	*NCOA7*	*ZNF2*		*OTX2*	
	*RSPO3*	*EPHB6*	*MTERFD2*	*VLDLR*		*NPTN*	
	*ANKRD13A*	*MLLT3*	*ZBTB41*	*CUGBP2*		*EIF2C1*	
	*PRDM2*	*NR3C1*	*NAPEPLD*	*SAR1B*		*SYNJ1*	

## Discussion

In the present study, we have measured the expression of plasma miRNAs and performed a characterization in a group of workers with chronic lead exposure. Our study identified six miRNAs that might be potentially lead related. By retrospective investigation and further validation, we finally identified that *miR-211* was strongly associated with lead exposure susceptibility. These findings suggested that *miR-211* could be regarded as a potential biomarker for personnel screening of lead-associated jobs.

Chronic lead poisoning is a complex occupational disease, which is considered as the consequence of interaction between genetics and environmental factors. The hazards of chronic lead poisoning include anemia [17,18], renal interstitial fibrosis [19,20], depression, and even Alzheimer’s disease [21]. Also, cancer mortality increased under lead exposure, which was reported in previous researches in larger populations [22,23], especially in female colon and rectal cancer patients [24]. As an imperative part of epigenetic factor, variations of miRNAs are closely related to lifestyle, age, ethnicity, environmental changes, and exposure to toxic substances. Expressions of *miR-525-5p, miR-527, miR-532-3p, miR-548*, and *miR-199a-5p* reduced in HEK293 cells after lead sulphide treatment. The intensity of comet tail in the same treated cells also revealed that DNA breaks arose, with miRNAs’ variations in human renal cell lines [25]. In our study, we detected that expressions of *miR-520c-3p, miR-211*, and *miR-148a* significantly differed in plasma between workers with minimal and high lead exposure.

Of these SNPs, *miR-520c-3p* had been reported to affect obesity [26]. In obesity research, there was a decreasing trend in *miR-520c-3p* from non-obese to morbidly obese patients [26]. Fat is considered to aggravate lead exposure in human body, which suggested the possible mechanism of *miR-520c-3p* in lead exposure. Besides, *miR-520c-3p* was also a functional miRNA for proliferation inhibiting in hepatocellular carcinoma by targetting GPC3 and eIF4GII [27,28], and the hepatic impact of lead might also be a source of *miR-520c-3p* in plasma. High profile of *miR-520c-3p* might weigh against liver recovery after lead exposure. Kidney is another widely known organ susceptible to lead poisoning. As Li et al. [29] reported, *miR-211* participated in the candidemia-induced kidney injuries via regulating HMX1 expression, and mimics of *miR-211* mitigated the kidney injuries, especially improving the renal glomerular filtration rate (GFR). In our study, *miR-211* was overexpressed in highly internal lead-exposed persons, who had a higher BLL. For this phenomenon, the epigenetic regulation of methylation was a plausible explanation. Another recent article demonstrated that DNMT1 could modulate the DNA methylation in the promoter region of *miR-211* and influence the expression of *miR-211* [30]. Surprisingly, there was a negative regulatory feedback loop between *miR-148a* and DNMT1: high profile of *miR-148a* could suppress the expression of DNMT1, but high expression of DNMT1 could improve the expression of *miR-148a* [31]. In our study, *miR-211* and *miR-148a* acted in a positive relationship, which suggested that methylation also took part during lead exposure in human body.

Some limitations of the present study existed as follows. First and foremost, misclassification was a potential problem. BLL records in our study were based on a one-time measurement during annual physical examination. Second, the half-life of lead in human body was relatively short, approximately 30 days [32]; thus the bone lead level should be a more appropriate choice for chronic lead exposure, which could usually sustain for 5–19 years [33]. Third, release of bone lead usually increased along with age, resulting in higher BLL in elder participants. In our study, participants in the high lead-exposure group were indeed older. Considering this pitfall, we adjusted age for analysis of miRNAs expression. In addition, the sample size was limited; and larger sample sizes with more detailed information are desirable for future studies.

In conclusion, our study is the largest of differential miRNA expression in lead-related workers. We were the first to report that miR-was to be associated with lead exposure, which could also be a potential predictive biomarker for lead susceptibility.

## Abbreviations

BLL: blood lead level
CDC: Center for Disease Control and Prevention
qRT-PCR: quantitative reverse-transcriptase PCR
IARC: International Agency for Research on Cancer
TWA: time-weighted average
SNP: single nucleotide polymorphism
KEGG: Kyoto Encyclopedia of Genes and Genomes
